# Polyphenolic Profile and Antioxidant Capacity of Extracts from *Gordonia axillaris* Fruits

**DOI:** 10.3390/antiox8060150

**Published:** 2019-05-29

**Authors:** Ya Li, Shi-Yu Cao, Sheng-Jun Lin, Jia-Rong Zhang, Ren-You Gan, Hua-Bin Li

**Affiliations:** 1 Guangdong Provincial Key Laboratory of Food, Nutrition and Health, Department of Nutrition, School of Public Health, Sun Yat-Sen University, Guangzhou 510080, China; liya28@mail3.sysu.edu.cn (Y.L.); caoshy3@mail2.sysu.edu.cn (S.-Y.C.); 2 Zhongshan Center for Disease Control and Prevention, Zhongshan 528403, China; zscdclsj@163.com; 3 Department of Food Science & Technology, School of Agriculture and Biology, Shanghai Jiao Tong University, Shanghai 200240, China; zhangjiarong@sjtu.edu.cn

**Keywords:** *Gordonia axillaris*, ultrasonic-assisted extraction, antioxidant, phenolic compound, response surface methodology

## Abstract

An ultrasonic-assisted extraction (UAE) method was adopted to extract natural antioxidants from edible *Gordonia axillaris* fruit. Single-factor experiments and response surface methodology were conducted to investigate the influences of five different parameters on antioxidant capacity. The optimal conditions of the UAE were 39.78% ethanol, 30.94 mL/g solvent/material ratio, 59.47 min extraction time, 40 °C temperature, and 400 W ultrasonication power. The antioxidant capacity was 525.05 ± 14.34 µmol Trolox/g DW under the optimal conditions, which was in agreement with the predicted one (531.71 µmol Trolox/g DW). Additionally, in comparison with two traditional methods (maceration and Soxhlet extraction), the established UAE method greatly improved the yield of antioxidants and significantly reduced the extraction time. Besides, nine phenolic compounds were identified and quantified in the extract of *Gordonia axillaris* fruits by ultra-performance liquid chromatography-tandem mass spectrometry (UPLC-MS/MS), including rutin, gallic acid, protocatechuic acid, epicatechin, 2-hydrocinnamic acid, *p*-coumaric acid, quercetin, epicatechin gallate, and ferulic acid. The richness of phenolic compounds in the *Gordonia axillaris* fruits indicated its potential health benefits, and its extract rich in antioxidants could be developed into functional food or nutraceuticals with the potential to prevent certain diseases induced by oxidative stress, such as cardiovascular diseases and cancers. This study also provided a way to enhance the economic values of *Gordonia axillaris* fruits compared to raw fruits.

## 1. Introduction

**Figure 1:**
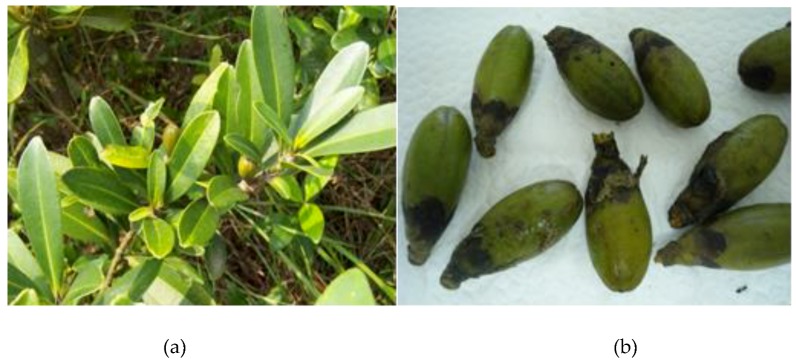
(**a**) The trees of *Gordonia axillaris*; (**b**) The fruits of *Gordonia axillaris*.

Many fruits have been found rich in phenolic compounds, which have been reported to possess antioxidant, antimicrobial, and anti-inflammatory activities [1,2]. *Gordonia axillaris* is widely distributed in southern America and China, such as Hong Kong, Sichuan, Yunnan, Hainan, and Guangxi provinces [3]. The trees of *Gordonia axillaris* are evergreen shrubs or small tree plants which can grow up to eight meters (Figure 1a), and are often planted as garden tree due to their attractive appearance. The different parts of *Gordonia axillaris*, including fruit, leaf, and stem bark, have been used in the traditional folk medicine for the treatment of certain ailments, such as stomachaches and diarrhea [4,5]. In addition, their anticancer and hepatoprotective activities have also been reported [3,6]. The fruit of *Gordonia axillaris* is an edible wild fruit, mainly used in herbal medicine. The fruit appears as a rough oval with a length about 2 cm and a width about 1 cm (Figure 1b). In our previous study, the fruits of *Gordonia axillaris* were found to possess strong antioxidant capacity with TEAC value 344 ± 12.1 μmol Trolox/g, TPC value 24.6 ± 1.08 mg GAE/g, FRAP value 180 ± 11.2 μmol Fe(II)/g based on tetrahydrofuran and methanol-acetate acid-water (50:3.7:46.3, v/v/v) extraction [7], suggesting that the *Gordonia axillaris* fruits can be a potential rich source of natural antioxidants. Furthermore, cultivation of this tree has increased in recent years due to the increasing economic and health potentials of the tree and its fruit. Since the extraction efficiency is the guarantee to recover bioactive components from the fruits, developing an efficient method to extract antioxidants, especially the phenolic compounds, from *Gordonia axillaris* fruit is critical for its value-added utilization.

A microwave-assisted extraction method was developed to extract antioxidants from this fruit previously [8]. Ultrasonic-assisted extraction (UAE) is another green and efficient extraction technique [9,10]. Thus, it is necessary to develop an alternative extraction method using UAE to extract antioxidants from this fruit, and to compare its extraction efficiency with conventional methods. Generally, the mechanisms of UAE have been mainly considered as a result of cavitation phenomena occurring during ultrasonic irradiation [11,12]. Recently, it has been pointed out that UAE works through various independent or combined mechanisms between fragmentation, capillarity, erosion, sonoporation, and detexturation [13]. In comparison with some conventional extraction methods such as maceration and hot reflux extraction, UAE has several advantages such as shorter extraction time, reduced organic solvent consumption, and less energy costs [14,15]. Therefore, UAE has been utilized to extract many bioactive components from natural products such as fruits [16,17]. 

During the UAE procedure, the extraction efficacy would be influenced by several parameters, such as the solvent, extracting time, and temperature [13]. These parameters could also interact with each other, affecting the extraction efficacy in a more complex way. Therefore, it is important to evaluate the interactions among these parameters, and response surface methodology (RSM) could be adopted to optimize the parameters and obtain the maximum yields of target compounds [18,19,20].

Compared to the raw fruit, the content of bioactive components in the extract could be increased obviously, and the volume and weight of the extract could be decreased markedly. Therefore, the effective concentration of bioactive compounds could be reached more easily by intake of the extract than consumption of the raw fruit. The present study is aimed at establishing an efficient UAE method to extract natural antioxidants from the *Gordonia axillaris* fruits. Moreover, ultra-high-performance liquid chromatography coupled with tandem mass spectrometry (UPLC-MS/MS) was adopted to identify and quantify main phenolic compounds in the extract. Compared to the raw fruits, *Gordonia axillaris* fruit extracts can be concentrated in natural antioxidants, which can be used as high-value functional ingredients or additives to formulate different functional foods or nutraceuticals with the potential to prevent certain diseases related to oxidative stress. 

## 2. Materials and Methods

### 2.1. Chemicals and Reagents

The Folin–Ciocalteu’s phenol reagent, standard phenolic compounds (2-hydrocinnamic acid, rutin, daidzein, equol, coffeic acid, *p*-coumaric acid, epigallocatechin, quercetin, ferulic acid, epicatechin, glycitein, resveratrol, chlorogenic acid, gallic acid, epicatechin gallate, genistein, and protocatechuic acid), ABTS (2,2’-azinobis(3-ethyl-benothiazoline-6-sulphonic acid) diammonium salt), and Trolox (6-hydroxy-2,5,7,8-tetramethylchromane-2-carboxylic acid) were purchased from Sigma-Aldrich (St. Louis, MO, USA). Potassium acetate, potassium persulphate, aluminum chloride hexahydrate, and sodium carbonate were purchased from Tianjin Chemical Factory (Tianjin, China). The formic acid and methanol of HPLC grade were used for the UPLC-MS/MS analysis. Besides, all other chemicals and reagents used in this work were of analytical grade, and deionized water was used in all the experiments.

### 2.2. Sample Preparation

The ripe *Gordonia axillaris* fruits with overall the same maturity stage were manually collected from different *Gordonia axillaris* trees at the Lung Fu Mountain, Sai Wan, Hong Kong Island, Hong Kong, in August 2017. The fruit was ground into fine particles by a pulverizer (model XT-A400, Hongtaiyang Co., Ltd., Yongkang, China), freeze-dried, sieved (0.300 mm particle size), and then stored at 4 °C in a refrigerator.

### 2.3. Instruments

The extraction of antioxidants from *Gordonia axillaris* fruit was carried out in an ultrasonic bath device (Kj1012B, Guangzhou Kejin Ultrasonic Instrument Factory, Guangzhou, China). The device was equipped with a digital control system for working time, temperature, and irradiation power, with an ultrasonic frequency of 28 kHz, and the temperature is controlled using a water bath with a thermocouple in this device.

### 2.4. Extraction of Antioxidants

#### 2.4.1. Ultrasonic-Assisted Extraction

The powder of *Gordonia axillaris* fruits (0.500 g) was accurately weighed and mixed with a certain volume of ethanol-water solution in a tube, with the concentration based on the experimental design. Then, the tubes were immersed into a sonication water bath, and irradiated under different temperatures (°C), ultrasonication powers (W), and extraction time (min), as defined by the experimental design. After the extraction, the mixture was centrifuged (4200 × *g*, 15 min, and 4 °C), and the supernatant was collected and stored at 4 °C for further use. Additionally, the supernatant was filtered (0.45 µm membrane) prior to UPLC-MS/MS analysis.

#### 2.4.2. Maceration Extraction

The powder of *Gordonia axillaris* fruits (0.500 g) was extracted with 15.47 mL of 39.78% ethanol for 24 h at room temperature (25 °C) in a shaking water bath. After the extraction, the mixture was centrifuged (4200 × *g*, 15 min, and 4 °C), and the supernatant was collected for further analysis.

#### 2.4.3. Soxhlet Extraction

The Soxhlet extraction was conducted according to the method reported previously [15]. The powder of *Gordonia axillaris* fruits (0.500 g) was wrapped with Whatman filter paper and extracted with 400 mL of ethanol aqueous solution (39.78%) in a Soxhlet apparatus. After 4 h of extraction under 85 °C (maintained by a water bath), the obtained solution was collected for the subsequent analysis.

### 2.5. Determination of Antioxidant Capacity

Trolox equivalent antioxidant capacity (TEAC) assay was used to evaluate the antioxidant capacity of the extracts from the *Gordonia axillaris* fruits, and the procedure was carried out according to the literature [21]. In short, the ABTS stock solution was prepared by mixing 2.45 mmol/L potassium persulfate solution and 7 mmol/L ABTS^•+^ solution (1:1, v/v), and placed in the dark for 16 h before use. To test the sample, the ABTS working solution was prepared by diluting the ABTS stock solution with deionized water to make the absorbance at 0.70 ± 0.05 at 734 nm. Subsequently, a 0.1 mL sample was mixed with 3.8 mL diluted ABTS working solution for 6 min, and then the absorbance of the mixture was tested using a spectrophotometer (Model 722, Shanghai precision instrument Co., Ltd., Shanghai, China). Trolox was used as the reference compound, and the TEAC results were expressed as µmol Trolox/g dry weight (DW) of *Gordonia axillaris* fruits.

### 2.6. Determination of the Yield of Phenolic Compounds

In this study, the yield of phenolic compounds was determined by evaluating the total phenolic contents (TPC) in extracts. The TPC was determined based on the method reported in the previous literature [22]. Briefly, a 0.5 mL diluted sample was mixed with 2.5 mL 0.2 mol/L Folin-Ciocalteu reagent for 4 min, and then 2 mL saturated sodium carbonate solution (75 g/L) was added into the solution. After 2 h of incubation at room temperature, the absorbance of the solution was measured at 760 nm. Gallic acid was used as the reference compound, and the results of TPC were displayed as mg gallic acid equivalent (GAE)/g DW of *Gordonia axillaris* fruits. 

### 2.7. Determination of the Yield of Flavonoid Compounds

The total flavonoid content (TFC) in the extract was evaluated according to the method reported in the previous literature [23]. In brief, a 0.5 mL sample was added to the solution containing 1.5 mL ethanol (95%, v/v), 0.1 mL AlCl_3_ solution (10%, w/v), 0.1 mL potassium acetate solution (1 mol/L), and 2.8 mL deionized water. The solution was incubated for 30 min before testing the absorbance at 415 nm. Quercetin was used as the reference compound, and the results of TFC were expressed as mg quercetin equivalent (mg QE)/g DW of *Gordonia axillaris* fruits.

### 2.8. Analysis of Phenolic Compounds by UPLC-MS/MS

The phenolic compounds in the extracts obtained under the optimal condition of UAE were analyzed based on the method described in the previous literature with minor modifications [17]. An AB SCIEX 4000 QTRAP LC-MS/MS system (SCIEX, Framingham, MA, USA) was employed, equipped with an Acquity UPLC^®^ HSS T3 column (3.0 × 150 mm, 1.8 µm) (Waters, Milford, MA, USA) for the separation of phenolic compounds at 40 °C, and the injection volume was 2 µL. The mobile phase consisted of solution A (0.2% formic acid aqueous solution) and solution B (methanol), and the flow rate was 0.3 mL/min. The gradient elution was performed as follows: 0–2 min, 15% (B); 2–8 min, 15–30% (B); 8–15 min, 30–80% (B); 15–17.5 min, 80% (B); 17.5–19.5 min, 15% (B). For the conditions of MS, ESI source with the negative mode was selected and ion source temperature was 550 °C. The capillary voltage was −4,500 V and multiple reaction monitoring (MRM) mode was selected. The curtain gas, nebulizer gas, and auxiliary gas was 12, 20, and 20 psig, respectively. The phenolic compounds were tentatively identified by MS/MS, and further verified and quantified by comparing the retention time and peak area with those of the standards. The contents of phenolic compounds were expressed as µg/g DW.

### 2.9. Experimental Design

#### 2.9.1. Single-Factor Experiments

Single-factor experiments were carried out to evaluate the effects of five experimental parameters on the extraction efficacy, including ethanol concentration (20–80%), solvent/material ratio (S/M ratio, 10–60 mL/g), extraction time (0–90 min), extraction temperature (30–70 °C), and ultrasonication power (300–800 W). Based on the results from single-factor experiments, three factors showing major influences on the extraction efficacy would be selected in the subsequent response surface method design.

#### 2.9.2. Response Surface Methodology (RSM)

The RSM was conducted through a central composite rotatable design (CCRD) to optimize the yield of antioxidants from the *Gordonia axillaris* fruits. Based on the results of single-factor experiments, three independent variables, including ethanol concentration (X1, %), S/M ratio (X2, mL/g), and extraction time (X3, min), were selected for the RSM design. Three independent variables and their related codes and levels are displayed in Table 1. A three-factor, five-level CCRD with 20 experimental runs were carried out, including six replicates in the central point (Table 2). The response values of the 20 different runs were fitted to the following second-order polynomial equation:

(1) $$Y=\beta_{0}+\sum^{\;}\beta_{i}X_{i}+\sum^{\;}\beta_{\mathrm{ii}}X_{i}^{2}+\sum^{\;}\beta_{\mathrm{ij}}X_{i}X_{j}.$$

In the equation, X_i_, and X_j_ stand for independent variables. β_0_, β_i_, β_ii_, and β_ij_ (i ≠ j) stand for the regression coefficients for intercept, linear, quadratic, and interaction terms, respectively. 

### 2.10. Statistical Analysis

In this study, all the experiments were performed in triplicate, and the results were expressed as mean value ± standard deviation (SD). The statistical analysis was carried out using Excel 2016 (Microsoft, Redmond, WA, USA) and Design Expert (version 8.0.6, Stat-Ease, Minneapolis, MN, USA). Multiple comparisons were carried out by one-way analysis of variance (ANOVA) plus post hoc Tukey test. *P* < 0.05 was defined as statistical significance.

## 3. Results and Discussion

### 3.1. Results from Single-Factor Experiments 

**Figure 2:**
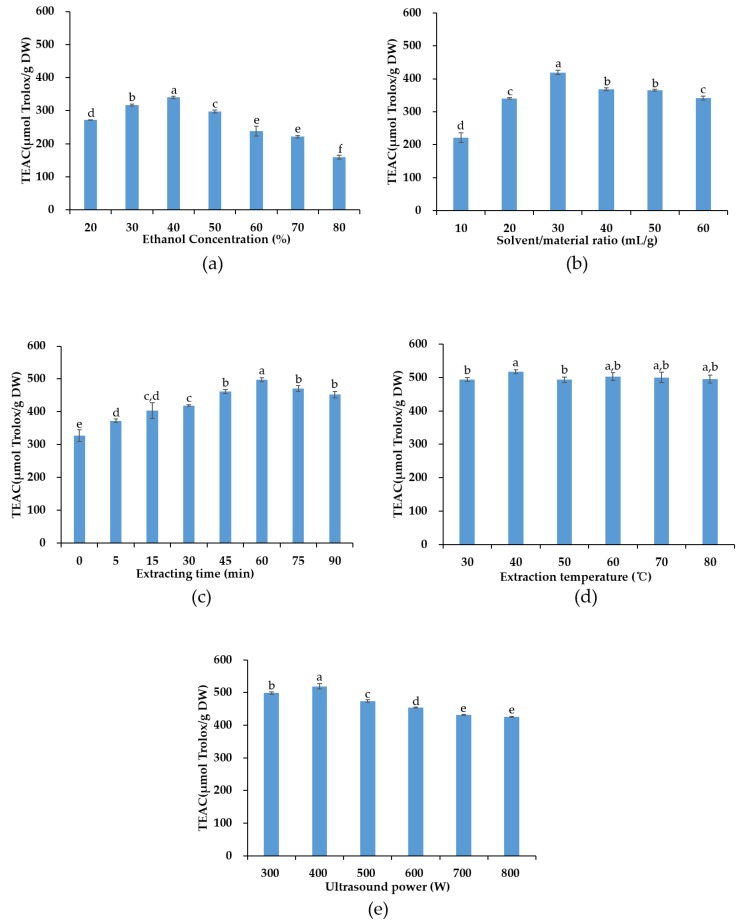
Effects of ethanol concentration (**a**), solvent/material ratio (**b**), extraction time (**c**), extraction temperature (**d**), and ultrasonication power (**e**) on the extraction efficacy. Different lowercase letters indicated statistical significance (*P* < 0.05). TEAC, Trolox equivalent antioxidant capacity.

Aqueous ethanol solution has been extensively used to extract natural bioactive compounds due to its low toxicity and easy accessibility [24,25]. In Figure 2a, the effects of different ethanol concentrations (20–80%) on the extraction efficiency were studied, with other extraction parameters fixed as follows, 20 mL/g S/M ratio, 30 min extraction time, 30 °C extraction temperature, and 400 W ultrasonication power. The yield of antioxidants increased following the increase of ethanol concentration from 20 to 40%, reached its peak at ethanol concentration of 40%, and then gradually decreased with the ethanol concentration from 40% to 80%, which could be due to the polarity of the mixture solvent that did not match with the polarity of the extract. Similar results were found in the study using UAE to extract antioxidants from the *Osmanthus fragrans* flower [26] and lycopene from papaya processing waste [27]. Therefore, 40% ethanol concentration was selected in the following experiments.

The effects of different S/M ratio (10–60 mL/g) on the TEAC value were investigated under the following conditions: 40% ethanol concentration, 30 min extraction time, 30 °C extraction temperature, and 400 W ultrasonication power. The results are presented in Figure 2b. The yield of antioxidants increased from 221.28 ± 15.28 to 419.01 ± 6.32 µmol Trolox/g DW as the S/M ratio rising from 10 mL/g to 30 mL/g. A possible explanation is that higher S/M ratio accelerated mass transfer and the diffusion of antioxidants into the solvent, thus increasing the yield of antioxidants [28,29]. However, a declining trend was observed when the S/M ratio raised from 30 mL/g to 60 mL/g, probably be due to the polarity difference between the mixture (including solvent and solute) and the substances to be extracted. Therefore, the S/M ratio of 30 mL/g was selected for the subsequent experiments.

The influences of different extraction time on the extraction efficacy were investigated in the range of 0–90 min, with other parameters fixed as follows, 40% ethanol concentration, 30 °C extraction temperature, 30 mL/g S/M ratio, and 400 W ultrasonication power. In Figure 2c, as the extraction time increased from 0 to 60 min, the TEAC value raised from 327.86 ± 18.09 to 497.88 ± 6.01 µmol Trolox/g DW. The maximum yield of antioxidants was obtained at 60 min. When the extracting time further increased from 60 to 90 min, the TEAC value began to decrease. The results suggested that in a certain range the increase in the ultrasonication time could accelerate to dissolve the target compounds, while prolonged ultrasonic irradiation time might induce to degrade some bioactive compounds. For instance, it was reported that after 80 min of UAE, the extraction yield of rutin from *Artemisia selengensis* Turcz decreased by about 13% compared with 50 min of UAE [30]. Similar results were also found in the study of extracting oleanolic acid and ursolic acid from *Ligustrum lucidum* Ait using UAE [31]. Therefore, the extracting time of 60 min was chosen for the following experiments.

Figure 2d illustrates the influences of extraction temperature (30–80 °C) on the yield of antioxidants. The rest parameters were fixed at 40% ethanol concentration, 30 mL/g S/M ratio, 60 min extraction time, and 400 W ultrasonication power. The yield of antioxidants increased as the increase of temperature, and reached its maximum at the temperature of 40 °C, which was 516.94 ± 5.93 µmol Trolox/g DW. When the temperature exceeded 40 °C, the yield of antioxidants dropped down, probably due to the degradation of some heat labile compounds at higher temperature. Consistently, it was reported that when extracting phenolic compounds from peel of *Citrus unshiu* by UAE, excessively high temperature caused decrease in the contents of caffeic acid, ferulic acid, *p*-coumaric acid, and *p*-hydroxybenzoic acid by 48.90, 48.23, 44.20, and 35.33%, respectively [32]. Therefore, 40 °C extraction temperature was selected for the following experiments.

Ultrasonication power could also affect the efficacy of UAE. In this section, the influences of ultrasonication power on the yield of antioxidants were evaluated in the range of 300–800 W, with other parameters fixed at ethanol concentration of 40%, S/M ratio of 30 mL/g, extraction time of 60 min and extraction temperature of 40 °C. The results are shown in Figure 2e. When ultrasonication power increased from 300 to 400 W, the yield of antioxidants increased, and the yield of phenolic compounds reached the maximum at the ultrasonication power of 400 W, and then gradually decreased. A possible explanation is that high ultrasonication power could induce the cavitation phenomena, the formation and collapse of more bubbles, thereby elevating the yield of target compounds. However, excessively strong ultrasonication power might decompose some compounds in the extracts, causing a lower yield of target compounds [33,34]. Consequently, the ultrasonication power of 400 W was selected in the experiments. 

### 3.2. Results of Response Surface Methodology Experiments

#### 3.2.1. Central Composite Rotatable Design (CCRD) and Results

**Figure 3:**
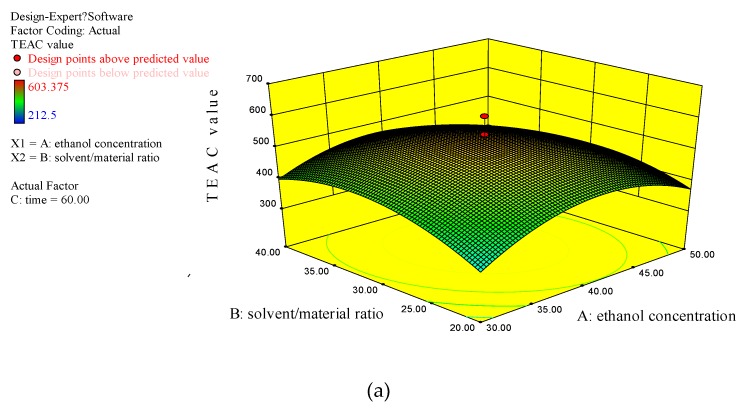

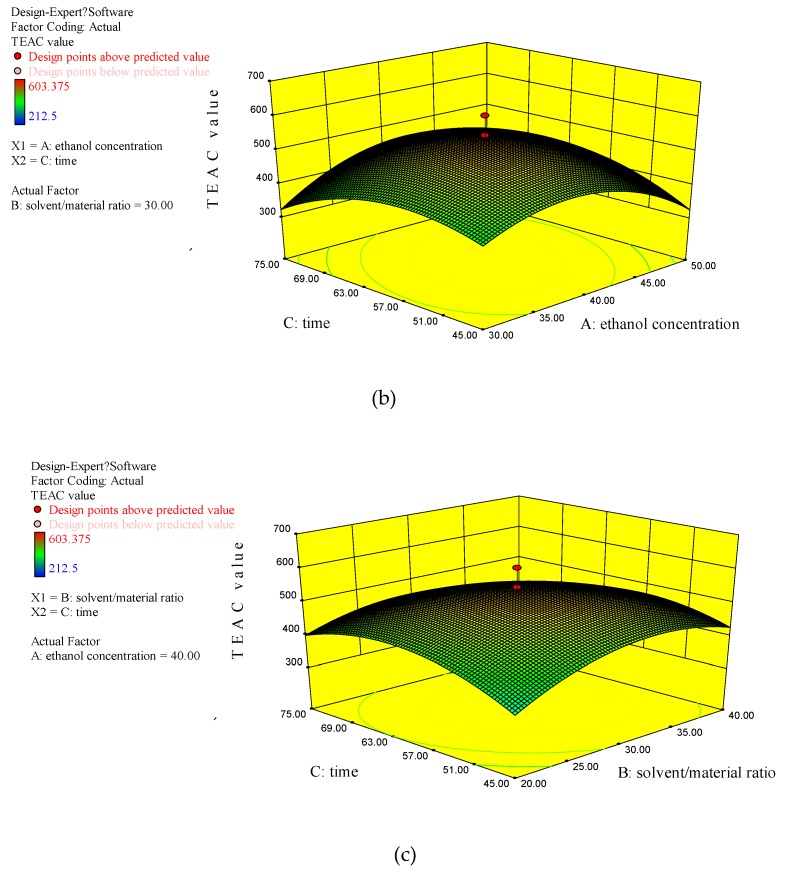
The three-dimensional plots of response surface. (**a**) The combined effects of ethanol concentration and solvent/material ratio (S/M) ratio when the extraction time was fixed at 60 min; (**b**) The interaction between ethanol concentration and extraction time when S/M ratio was fixed at 30 mL/g; (**c**) The combined effect of extraction time and S/M ratio when the ethanol concentration was fixed at 40%. TEAC, Trolox equivalent antioxidant capacity.

According to the single-factor experimental results, ethanol concentration, S/M ratio, and extraction time had greater effects on the extraction efficacy. Therefore, they were further investigated for their possible interaction (Figure 3) by response surface methodology to optimize the extraction efficacy. The middle level of each parameter was fixed as follows, 40% ethanol concentration, 30 mL/g S/M ratio, and 60 min extraction time, while the extraction temperature and ultrasonication power were fixed at 40 °C and 400 W, respectively. The experimental design, experimental values, and predicted values of the 20 runs are shown in Table 2. The TEAC value varied from 212.50 to 603.38 µmol Trolox/g DW. 

#### 3.2.2. Fitting the Model

The data in Table 2 were analyzed using multiple regression fitting, and a quadratic polynomial regression model equation (2) describing the relationship between the response value and the three variables was generated:

(2) $$Y=531.11-0.62X_{1}+12.07X_{2}-1.82X_{3}-23.67X_{1}X_{2}+38.29X_{1}X_{3}-26.85X_{2}X_{3}\;-96.61X_{1}^{2}-72.26X_{2}^{2}-72.34X_{3}^{2}.$$

Results of ANOVA is displayed in Table 3. The high *F* value (*F* = 15.02) and low *p* value (*p* = 0.0001) of the model, and the low *F* value (*F* = 1.74) and high *p* value (*p* = 0.2791) of “lack of fit” indicated the reliability of the generated model. In addition, the high determination coefficient value (R^2^) of 0.9311, and the adjusted R^2^ value (Adj. R^2^) of 0.8691 suggested that the regression model could explain 93.1% of the response value variations.

#### 3.2.3. Analysis of Response Surface Plots

The three-dimensional plots of response surface (Figure 3) illustrated the interaction of two of the three major factors on the yield of antioxidants when the third one was fixed at a certain level. Figure 3a shows the combined effect of ethanol concentration and S/M ratio when the extraction time was fixed at 60 min. Both ethanol concentration and S/M ratio had effects on the yield of antioxidants. The yield of antioxidants gradually increased when the ethanol concentration increased in a certain range, and when the ethanol concentration exceeded about 40%, the yield of antioxidants began to decrease. As for the S/M ratio, the yield of antioxidants increased at low S/M ratio (20–35%), and gradually decreased when the S/M ratio further increased. The interaction between ethanol concentration and extraction time is shown in Figure 3b. The yield of antioxidants changed with the ethanol concentration or extraction time when the S/M ratio was fixed at 30 mL/g. In Figure 3b, a similar influence of ethanol concentration on the response value was observed. As for the extraction time, the TEAC value gradually increased as the extracting time rising. When the extracting time exceeded nearly 60 min, the TEAC value gradually decreased. In Figure 3c, the S/M ratio and extracting time also showed similar effects on the response value with that shown in Figure 3a,b. Combining the response surface plots and the ANOVA results in Table 3, conclusions could be drawn that the effect of each independent variable on the response value was quadratic. In addition, the S/M ratio had a more significant effect on the response value than the ethanol concentration and extracting time.

#### 3.2.4. Verification of the Predicted Value

The generated quadratic polynomial regression model gave the optimal extraction conditions with a desirability of 0.663. The optimal extraction conditions were as follows: 39.78% ethanol concentration, 30.94 mL/g S/M ratio, 59.47 min extraction time, 40 °C temperature, and 400 W ultrasonication power. The yield of antioxidants under the optimal conditions was predicted to be 531.71 µmol Trolox/g DW. In order to verify the model’s ability to accurately predict the actual value, verification experiments (six replicates) under the optimal conditions were carried out, and the result was 525.05 ± 14.34 µmol Trolox/g DW, which was close to the predicted value.

### 3.3. Comparison of UAE with Two Conventional Extraction Methods

Maceration and Soxhlet extraction are two conventional extraction methods which are commonly used to extract bioactive components from natural products [35,36]. Therefore, the efficacy of UAE was compared with that of maceration and Soxhlet extraction, and the results are displayed in Table 4. The TEAC value of UAE procedure was 2.26 times higher than that of maceration. Meanwhile, the UAE required much shorter time (59.47 min vs. 24 h), and the TPC and TFC of the extract obtained by UAE were both higher than those obtained by maceration. In addition, compared with the Soxhlet extraction, the UAE enhanced the extraction efficacy by 3.62 times, with a lower temperature (40 °C vs. 85 °C) and shorter time (59.47 min vs. 4 h). In addition, UAE also increased the yield of phenolic compounds and flavonoids compounds from the *Gordonia axillaris* fruit. Similar results were reported to extract antioxidants from the flower of *Limonium sinuatum*, in which the established UAE method extracted significantly higher content of antioxidants than Soxhlet extraction [37]. Another study also found that 10 min of ultrasound-assisted extraction of phenolic-rich heteroxylans from wheat bran yielded total polysaccharides similar to 60 min of conventional extraction, indicating that UAE reduced the process time [38]. In addition, UAE improved the extraction efficiency with shorter extraction time and higher TEAC, TPC, and TFC values for *Gordonia axillaris* fruits compared with microwave-assisted extraction (MAE), with the extraction time of 71.04 min, TEAC value of 198.16 ± 5.47 µmol Trolox/g DW, TFC value of 17.69 ± 1.02 mg GAE/g DW, and TPC value of 3.11 ± 0.12mg QE/g DW [8].

### 3.4. Phenolic Compounds in Extracts

**Figure 4:**
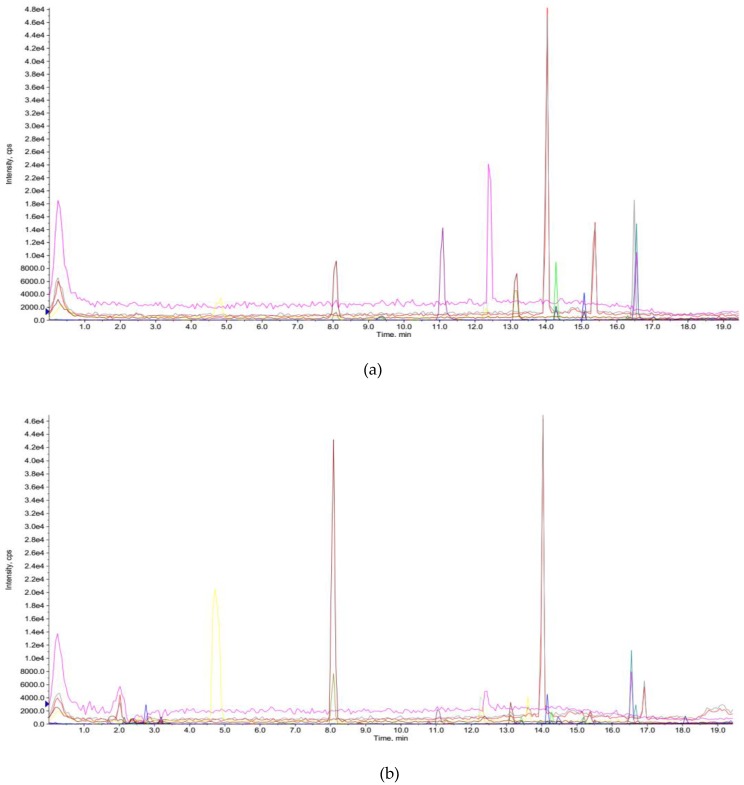
(**a**) The total ion chromatograms of standard compounds; (**b**) The total ion chromatograms of the sample obtained under the optimal conditions. Different colors in the figure indicated different compounds.

UPLC-MS/MS was next used to characterize the phenolic profile in the extract of *Gordonia axillaris* fruit acquired under the optimal condition of UAE. The total ion chromatograms of standard compounds and the sample acquired under the optimal conditions are shown in Figure 4. Nine phenolic compounds were identified and quantified (Table 5). Rutin had the highest content (21.86 ± 3.38 µg/g DW), followed by gallic acid (17.80 ± 2.68 µg/g DW), protocatechuic acid (10.81 ± 2.54 µg/g DW), epicatechin (3.89 ± 0.43 µg/g DW), 2-hydrocinnamic acid (2.68 ± 0.25 µg/g DW), *p*-coumaric acid (2.67 ± 0.22 µg/g DW), quercetin (2.45 ± 0.21 µg/g DW), epicatechin gallate (1.07 ± 0.11 µg/g DW), and ferulic acid (0.12 ± 0.01 µg/g DW). The contents of phenolic compounds in this study based on UAE were found different compared to those reported in a previous paper using MAE [8], probably due to the different extraction conditions, such as the different extraction mechanisms and extraction time between UAE and MAE.

## 4. Conclusions

In this study, an ultrasonic-assisted extraction method was developed to extract natural antioxidant polyphenols from the fruit of *Gordonia axillaris*, and single-factor experiments, and response surface methodology were used to acquire optimal extraction parameters. A quadratic polynomial regression model was established, and the optimal conditions were as follows: ethanol concentration at 39.78%, S/M ratio at 30.94 mL/g, extraction time at 59.47 min, temperature at 40 °C, and ultrasonication power at 400 W. Under the optimal conditions, the yield of antioxidants was 525.05 ± 14.34 µmol Trolox/g DW. The high R^2^ (0.9311) and the consistency between the predicted value and actual value suggested that this model could accurately predict the effects of the three major factors on the yield of antioxidants. Additionally, the optimized UAE method was more efficient than two conventional methods in extracting antioxidants from *Gordonia axillaris* fruits. Furthermore, nine phenolic compounds were identified and quantified by UPLC-MS/MS in the extract obtained under the optimal conditions. These phenolic compounds may be responsible for the strong antioxidant capacity of *Gordonia axillaris* fruits. In the future, the extract of the fruits can be developed into functional food or nutraceuticals with the potential to prevent certain oxidative stress-related chronic diseases. 

## Figures and Tables

**Table 1:** Five levels of the three variables of the extraction process.

Variable	Units	Symbol	Coded Levels				
			−1.68	−1	0	1	1.68
Ethanol concentration	% (*v*/*v*)	X_1_	23.18	30	40	50	56.82
Solvent/material ratio	mL/g	X_2_	13.18	20	30	40	46.82
Extraction time	min	X_3_	34.77	45	60	75	85.23

**Table 2:** The experimental design, experimental value, and predicted value of RSM.

Run	X_1_ (Ethanol Concentration, %)	X_2_ (Solvent/Material Ratio, mL/g)	X_3_ (Extraction Time, min)	Y (TEAC, µmol Trolox/g DW)	
				Actual Value	Predicted Value
1 *	40.00	30.00	60.00	511.85	531.11
2	40.00	46.82	60.00	325.69	347.03
3	56.82	30.00	60.00	243.01	256.81
4	40.00	30.00	85.23	317.37	323.43
5	30.00	40.00	45.00	423.16	393.22
6	50.00	20.00	75.00	376.83	364.21
7	30.00	40.00	75.00	278.25	259.29
8	50.00	40.00	45.00	293.50	268.04
9	40.00	30.00	34.77	275.42	329.54
10	50.00	20.00	45.00	261.15	237.55
11 *	40.00	30.00	60.00	523.29	531.11
12 *	40.00	30.00	60.00	508.04	531.11
13	40.00	13.18	60.00	267.60	306.44
14	50.00	40.00	75.00	283.33	287.29
15 *	40.00	30.00	60.00	504.23	531.11
16	23.18	30.00	60.00	212.50	258.88
17	30.00	20.00	45.00	314.54	268.02
18 *	40.00	30.00	60.00	603.38	531.11
19 *	40.00	30.00	60.00	546.17	531.11
20	30.00	20.00	75.00	258.61	241.51

* stands for the six replicates of central point.

**Table 3:** ANOVA of the fitted polynomial quadratic model.

Source	Sum of Squares	df	Mean Square	*F* Value	*p* Value	Significant
Model	2.635 × 10^5^	9	29279.68	15.02	0.0001	significant
X_1_ (Ethanol concentration)	5.21	1	5.21	2.673 × 10^−3^	0.9598	
X_2_ (Solvent/material ratio)	1988.99	1	1988.99	1.02	0.3363	
X_3_ (Time)	44.99	1	44.99	0.023	0.8823	
X_1_X_2_	4484.03	1	4484.03	2.30	0.1604	
X_1_X_3_	11730.86	1	11730.86	6.02	0.0341	
X_2_X_3_	5768.58	1	5768.58	2.96	0.1162	
X_1_^2^	1.345 × 10^5^	1	1.345 × 10^5^	68.98	<0.0001	
X_2_^2^	75240.48	1	75240.48	38.59	<0.0001	
X_3_^2^	75423.11	1	75423.11	38.68	<0.0001	
Residual	19499.88	10	1949.99			
Lack of Fit	12381.50	5	2476.30	1.74	0.2791	not significant
Pure Error	7118.37	5	1423.67			
Cor Total	2.830 × 10^5^	19				
R-Squared	0.9311					
Adj R-Squared	0.8691					

**Table 4:** The comparison of UAE with the maceration and Soxhlet extraction.

Extraction Methods	Ethanol Conc. (%)	Time	Temp. (°C)	TEAC (µmol Trolox/g DW)	TPC (mg GAE/g DW)	TFC (mg QE/g DW)
Maceration	39.78	24 h	25	161.25 ± 2.29	13.16 ± 2.43	1.86 ± 0.12
Soxhlet	39.78	4 h	85	113.77 ± 2.58	9.02 ± 0.78	1.77 ± 0.22
UAE	39.78	59.47 min	40	525.05 ± 14.34	43.45 ± 3.16	3.22 ± 0.44

TEAC, Trolox equivalent antioxidant capacity; TPC, Total phenolic contents; TFC, Total flavonoid contents; UAE, Ultrasonic-assisted extraction.

**Table 5:** The phenolic profile in the extract acquired under the optimal conditions of UAE.

Phenolic Components	Retention Time (*t*_R_, min)	Parent Ion (*m*/*z*, [M − H]¯)	Product Ion (*m*/*z*)	Contents (µg/g DW)
Rutin	14.77	609	300, 343	21.86 ± 3.38
Gallic acid	4.76	169.1	125, 112	17.80 ± 2.68
Protocatechuic acid	8.07	153.1	109, 108	10.81 ± 2.54
Epicatechin	12.3	289	203, 245	3.89 ± 0.43
2-Hydrocinnamic acid	14.03	163.1	119, 90	2.68 ± 0.25
*p*-Coumaric acid	14.03	162.7	119, 90	2.67 ± 0.22
Quercetin	16.54	301	179, 151	2.45 ± 0.21
Epicatechin gallate	13.13	441	169, 289.1	1.07 ± 0.09
Ferulic acid	14.28	193.1	134, 178	0.12 ± 0.01
